# Pulmonary arterial hypertension after congenital heart defect correction: a call for timely diagnosis and careful risk stratification to improve outcomes

**DOI:** 10.1093/ehjopen/oeaf172

**Published:** 2025-12-18

**Authors:** Qiangqiang Li, Yuan He, Andrew Constantine, Konstantinos Dimopoulos, Chen Zhang, Qiang Wang, Hong Gu

**Affiliations:** Department of Pediatric Cardiology, Beijing Anzhen Hospital, Capital Medical University, No. 2 Anzhen Road, Chaoyang District, Beijing 100029, China; Department of Pediatric Cardiology, Beijing Anzhen Hospital, Capital Medical University, No. 2 Anzhen Road, Chaoyang District, Beijing 100029, China; Adult Congenital Heart Disease Unit, Queen Elizabeth Hospital Birmingham, University Hospitals Birmingham NHS Foundation Trust, Mindelsohn Way, Edgbaston, Birmingham B15 2GW, UK; Department of Cardiovascular Sciences, University of Birmingham, Edgbaston, Birmingham B15 2TT, UK; Adult Congenital Heart Centre and National Centre for Pulmonary Hypertension, Royal Brompton Hospital, Royal Brompton & Harefield NHS Foundation Trust, Sydney Street, London SW3 6NP, UK; National Heart and Lung Institute, Imperial College London, Cale Street, London SW3 6LY, UK; Department of Pediatric Cardiology, Beijing Anzhen Hospital, Capital Medical University, No. 2 Anzhen Road, Chaoyang District, Beijing 100029, China; Department of Pediatric Cardiology, Beijing Anzhen Hospital, Capital Medical University, No. 2 Anzhen Road, Chaoyang District, Beijing 100029, China; Department of Pediatric Cardiology, Beijing Anzhen Hospital, Capital Medical University, No. 2 Anzhen Road, Chaoyang District, Beijing 100029, China

**Keywords:** Congenital heart disease, Pulmonary vascular disease, Survival, Pulmonary arterial hypertension, Prognosis

## Abstract

**Aims:**

Patients with pulmonary arterial hypertension (PAH) after congenital heart disease (CHD) correction (PAH-CHDcor) are becoming the most prevalent and rapidly expanding group within PAH associated with CHD (PAH-CHD), yet data on its presentation, long-term outcomes and prognostic variables are lacking. We report on a large paediatric and adult population with PAH-CHDcor, focusing on clinical presentation and long-term survival.

**Methods and results:**

We studied 127 PAH-CHDcor patients (mean age 21.5 ± 10.5 years; 74.8% female) diagnosed via cardiac catheterization from 2006 to 2022. The majority had post-tricuspid shunts (73.2%), with combined pre- and post-tricuspid (11.8%) and complex shunts (6.3%) less frequent. Pulmonary vascular resistance (PVR) at diagnosis averaged 13.2 ± 8.9 WU. Diagnosis occurred late (>5 years post-repair) in 43.3% of patients. Median follow-up was 4.0 (IQR 2.0–6.4) years. Kaplan-Meier estimates for survival at 3 and 5 years were 93.3% and 89.6%, respectively. Higher baseline PVR predicted mortality (HR 1.10, 95% CI 1.03–1.16, *P* = 0.003) and was the strongest multivariable predictor of a composite endpoint (death, heart failure hospitalization, or parenteral prostacyclin initiation; HR 1.11, 95% CI 1.05–1.18, *P* < 0.001). An exploratory application of a paediatric prognostic score (GOSH) showed excellent discriminative power for mortality (AUC 0.867) and the composite endpoint (AUC 0.856) at 5 years in this independent cohort.

**Conclusion:**

Mortality and morbidity are considerable in patients with PAH-CHDcor despite modern management. Regular, careful screening of all patients with repaired CHD is essential to ensure early diagnosis and risk stratification, with proactive evidence-based treatment to improve outcomes in this expanding population.

## Graphical Abstract

**Graphical Abstract:**
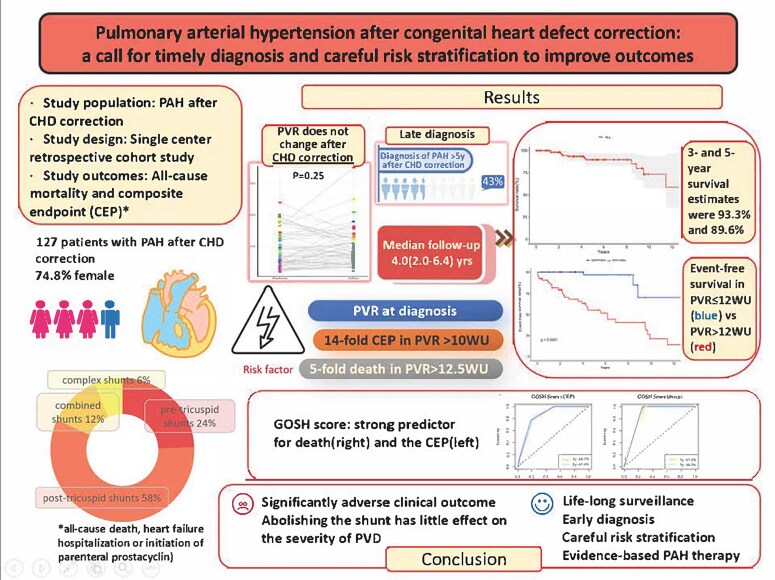

Key learning points What is already known

- Pulmonary arterial hypertension after congenital heart disease correction (PAH-CHDcor) is a growing clinical problem, increasingly recognized as survival after CHD repair improves worldwide.
- Prognosis in PAH-CHDcor is poor, often approximating that of idiopathic PAH and worse than Eisenmenger syndrome, despite the latter’s more severe pulmonary vascular disease.
- Early and late presentation after repair is common, but data on long-term outcomes and validated prognostic tools in age-agnostic PAH-CHDcor populations are limited.

What this study adds

- In a large Chinese cohort, PAH-CHDcor was associated with high medium- to long-term morbidity, with higher pulmonary vascular resistance at diagnosis emerging as the strongest independent predictor of adverse outcome.
- An exploratory application of the prognostic score from the UK National Paediatric PH Service (GOSH score) showed excellent discrimination for both mortality and the composite endpoint of death, heart failure hospitalization, or initiation of parenteral prostacyclin therapy in this independent population.
- These findings underscore the need for systematic, lifelong surveillance of all patients after CHD repair, timely invasive assessment when PAH is suspected, and proactive, risk-stratified use of PAH therapies to improve outcomes in this vulnerable group.

## Background

Pulmonary arterial hypertension (PAH) is a well-described, feared complication in patients with congenital heart disease (CHD), associated with excess mortality and morbidity.^1^ PAH is typically encountered in patients with unrepaired CHD, but is increasingly diagnosed following defect correction (PAH-CHDcor). In these patients, PAH is associated with an adverse outcome, which is similar to idiopathic PAH, but worse compared to adult patients with Eisenmenger syndrome, despite more severe pulmonary vascular disease and a large, unoperated defect in the latter.^2^ However, PAH-CHDcor is a heterogeneous population, with variable anatomy, timing of intervention, and pre-repair hemodynamics.^3–8^ Indeed, some patients with milder forms of PAH-CHDcor may remain clinically stable for years or decades, while others can progress rapidly, even in childhood.

Modern PAH therapies have significantly improved the prognosis of PAH patients and are, nowadays, widely used in PAH-CHDcor,^9,10^ yet limited data are available on the clinical presentation and determinants of medium-to-long-term prognosis of patients with PAH-CHDcor. While this population is expanding in the Western world, growth has been exponential in countries like China, where an aggressive strategy of delayed CHD correction had been adopted in previous decades.^11,12^ This provides an opportunity to study the clinical presentation and outcome of PAH-CHDcor in large cohorts of patients, and identify predictors of outcome, which are likely to be relevant to patients worldwide.

In this study, we examined a large cohort of paediatric and adult patients diagnosed with PAH-CHDcor and report on their clinical characteristics, timing of diagnosis, pre and post-closure hemodynamics and medium-to-long-term outcomes.

## Methods

### Study subjects

**Figure 1:**
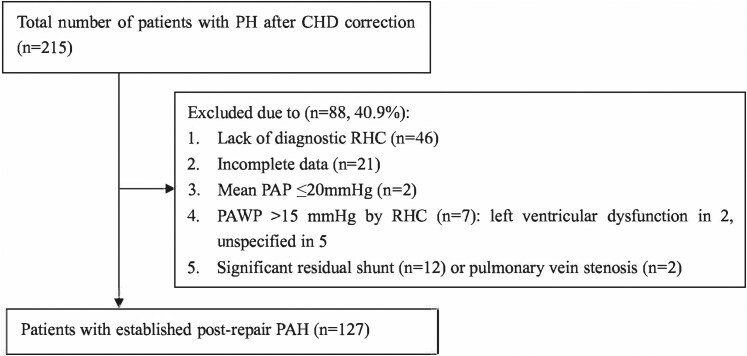
Study population and process of inclusion. PH: pulmonary hypertension; PAH: pulmonary arterial hypertension; CHD: congenital heart disease; RHC: right heart catheterization; PAP: pulmonary artery pressure; PAWP: pulmonary artery wedge pressure.

Patients diagnosed with PAH and fully anatomical corrected CHD in our tertiary center between September 2006 and June 2022 were included in this study (*Figure 1*). For the purpose of this study, PAH was defined as mean pulmonary arterial pressure >20 mmHg, pulmonary artery wedge pressure ≤15 mmHg and pulmonary vascular resistance (PVR) > 2 Wood Units (WU) (or indexed PVR＞3 WU·m^2^ for patients under 14 years old) by right heart catheterization (RHC).^13^ According to CHD types based on preoperative echocardiography, patients were classified into the following four groups: Simple pre-tricuspid shunts (atrial septal defect, ASD), simple post-tricuspid shunts (ventricular septal defect, VSD, or patent ductus arteriosus, PDA), combined simple shunts (any combination of VSD, PDA or ASD) and complex shunts (cardiac shunts combined with conus anomalies, anomalous pulmonary venous return, left heart obstructive defects including subaortic stenosis or coarctation of aorta). Patients with a significant residual shunt, single ventricle physiology after cavo-pulmonary anastomosis, segmental PH, lung disease, and other types of pulmonary hypertension (PH) were excluded from this study. A residual shunt was deemed significant when associated with a Qp/Qs >1.5 on cardiac catheterization and/or evidence of volume load. The methods used to collect and analyze data were approved by the Institutional Ethics Committee (reference number: 2023024X).

### Data collection

Patient data were retrospectively collected from the electronic medical record system. The date of RHC was used as the baseline for the survival analysis. Baseline clinical characteristics included patient demographics, CHD diagnosis, date and type of reparative operation or intervention, use of targeted PAH therapies, and laboratory parameters, including brain natriuretic peptide (BNP). RHC was performed in the catheterization lab with FiO2 < 0.3. Children under 14 years were sedated by a cardiac anaesthetist. Invasive haemodynamics, including mean pulmonary artery pressure (PAP), systemic arterial blood pressure, pulmonary artery wedge pressure, right atrial pressure, and oxygen saturations, were measured using a multipurpose or balloon-tipped catheter. Pressures were recorded at end-expiration and averaged over ≥3 cardiac cycles with the transducer zeroes at the mid-thoracic level. Waveforms were checked for damping and re-measured if necessary. Oxygen saturations were measured by co-oximetry. Cardiac output was determined by the indirect Fick method, and cardiac Index, PVR, Indexed PVR (PVRI), and the ratio of PVR to systemic vascular resistance were calculated. Stroke volume was calculated as cardiac output/heart rate, pulmonary artery compliance (PAC) as stroke volume/pulmonary pulse pressure (systolic—diastolic PAP). Right ventricle to pulmonary artery coupling was estimated by the tricuspid annular plane systolic excursion/systolic PAP ratio, when available. Echocardiographic data were also collected, including peak tricuspid regurgitation velocity on continuous wave Doppler, biventricular size and function, and valve function. Right ventricular dysfunction was assessed on a combination of qualitative and (when available) quantitative metrics, including TAPSE <16 mm or Z-score ≤ −2, FAC <35%, as per routine clinical practice. The echocardiographic probability of PH was judged from the TRV and supportive signs of right-heart pressure overload: new or worsening RV or RA dilatation or RV dysfunction (in the absence of an open/residual shunt), distended inferior vena cava with diminished inspiratory collapsibility and short pulmonary acceleration time. A TRV >2.8 m/s together with at least 1 supportive sign was considered suggestive of PH.

For the purpose of survival analysis, we used all-cause mortality and the composite endpoint of death, heart failure hospitalization or initiation of intravenous/subcutaneous prostanoids as outcome measures. Other adverse events were recorded, including syncope, hemoptysis, and symptomatic arrhythmia requiring intervention. Targeted PAH therapies included prostanoids, endothelin receptor antagonists or phosphodiesterase type-5 inhibitors. In this study, ‘treat-and-repair’ denotes targeted PAH therapy before defect closure, with intervention undertaken only if repeat RHC confirmed PVR <5 WU.

### Statistical analysis

All statistical analyses were performed using SPSS Statistics (IBM Corporation, Armonk, New York) and R version 4.2.2 (R Foundation for Statistical Computing, Vienna, Austria). Continuous variables are described as mean ± standard deviation or median (interquartile range), and categorical variables as number (percentage). The Kolmogorov-Smirnov test was used as a test for normality for a given variable. Differences between groups were assessed using the chi-square, *t*-test or Mann–Whitney U-test, as appropriable. One-way analysis of variance (ANOVA) or Kruskal-Wallis H tests examined differences among underlying CHD diagnoses. Kaplan-Meier curves were used to study survival rate. Comparisons between groups were made using the log-rank test. Cox regression analysis was used to identify predictors of mortality and the composite endpoint; the Schoenfeld residual test was used to verify the assumption of proportional hazards. Parameters associated with the composite endpoint on univariable analysis were included in a stepwise, multivariable Cox model. To avoid overfitting, multivariable Cox models adhered to a rule of 10 events per variable (35 composite events, hence ≤3 covariates entered). Age at CHD correction was analysed as a continuous covariate in Cox models. We also applied the score developed by Constantine et al. based on the UK National paediatric PH registry to our cohort as an exploratory analysis.^14^ This is an unweighted, additive score consisting of the following variables: absence of pre-operative PH, breathlessness (which we defined as World Health Organization functional class > I), presence of RV dysfunction on echocardiography (semi-quantitative), and a combined variable of post-repair PVRI >14 WU.m^2^ or high-risk anatomic lesion.^15^ We applied this score for the endpoints of a) all-cause mortality and b) the composite endpoint, as defined above, and used the package time ROC for time-dependent Receiver-Operating Characteristic analysis. A two-sided *P*-value <0.05 was considered statistically significant.

## Results

### Study population

A total of 127 patients were included in this study. Patient characteristics and clinical parameters at the time of diagnostic RHC are shown in *Table 1*. Average age was 21.5 ± 10.5 years and 95(74.8%) were female. There were 26(20.5%) children younger than 12 years and 43(41.7%) were under 18 years of age. Only 2(1.6%) patients had Down syndrome. Simple pre-tricuspid shunts had been diagnosed in 31(24.4%) patients, simple post-tricuspid shunts in 73(57.5%), and combined simple shunts in 15(11.8%). Eight (6.3%) patients had complex shunts, including PDA combined with interrupted aortic arch (*n* = 2), VSD combined with coarctation of aorta (*n* = 3), ASD combined with partial anomalous pulmonary venous drainage (*n* = 2) and PDA combined with right pulmonary artery from the ascending aorta (*n* = 1). The preoperative echocardiographic assessment had confirmed a nonrestrictive shunt in the vast majority (96.9%) of patients, while 3.1% patients had small defects, including 3 patients with an ASD <10 mm in diameter and 1 patient with a VSD of 5 mm in diameter combined with small PDA of 2 mm.

**Table 1:** Baseline characteristics and outcomes of patients with post-operative pulmonary arterial hypertension according to the type of congenital heart disease

	Overall (*n* = 127)	Simple pre-tricuspid shunt (*n* = 31)	Simple post-tricuspid shunt (*n* = 73)	Combined shunts (*n* = 15)	Complex shunts (*n* = 8)	*P*-value of difference between all groups	*P*-value of difference between simple pre-tricuspid shunt and post-tricuspid shunt
Age (years)	21.5 ± 10.5	28.2 ± 10.3	20.5 ± 9.0	15.8 ± 10.1	15.3 ± 13.2	<0.001	<0.001
Female, *n* (%)	95 (74.8)	29 (93.5)	49 (67.1)	12 (80)	5 (62.5)	0.03	0.003
Weight (kg)	47.7 ± 16.7	53.8 ± 15.5	48.6 ± 16.3	37.2 ± 14.6	35.4 ± 14.6	0.002	0.13
BSA (m^2^)	1.43 ± 0.32	1.53 ± 0.29	1.46 ± 0.31	1.23 ± 0.33	1.19 ± 0.37	0.02	0.06
WHO-FC, *n* (%)						0.55	0.01
I-II	105 (82.7)	30 (96.8)	55 (75.3)	12 (80)	8 (100)		
III-IV	22 (17.3)	1 (3.2)	18 (24.7)	3 (20)	0 (0)		
6MWD (m)	478.8 ± 115.7	520.7 ± 113.5	467.0 ± 114.7	420.5 ± 115.7	498.8 ± 70.7	0.07	0.04
Hb (g/L)	138.7 ± 18.4	133.0 ± 18.7	140.5 ± 18.3	144.0 ± 17.5	134.1 ± 15.9	0.14	0.06
BNP (pg/mL)	54 (27,134)	36 (25,51)	67 (33,162)	65 (51,87)	58 (23,85)	0.15	0.04
Echocardiographic parameters							
Peak TRV (m/s)	4.1 (3.4,4.8)	3.2 (2.9,4.0)	4.4 (3.8,5.0)	3.9 (3.4,5.0)	4.1 (3.7,4.8)	<0.001	<0.001
Ratio of RV/LV	1.03 (0.84,1.30)	0.90 (0.83,1.23)	1.05 (0.87,1.32)	1.08 (0.88,1.34)	0.91 (0.75,1.04)	0.16	0.10
Thickness of RV free wall (mm)	8 (7,10)	7 (6,8)	8.2 (7,10)	8.5 (7,10)	7 (6,8)	0.01	0.003
Ratio of TAPSE to systolic PAP^a^	0.22 (0.14,0.39)	0.39 (0.25,0.51)	0.19 (0.13,0.25)	0.20 (0.10,0.23)	—	0.01	0.003
Severe TVR, *n* (%)	19 (15.0)	4 (12.9)	11 (15.1)	4 (26.7)	0 (0)	0.92	0.77
Hemodynamic parameters							
SvO₂ (%)	69.1 ± 6.9	71.0 ± 5.3	68.3 ± 7.1	66.9 ± 8.5	73.4 ± 6.1	0.048	0.06
Systolic PAP (mmHg)	85.4 ± 35.8	61.8 ± 33.6	94.4 ± 33.2	94.8 ± 37.5	77.5 ± 22.1	<0.001	<0.001
Mean PAP (mmHg)	59.9 ± 25.5	41.9 ± 21.0	66.8 ± 24.3	66.5 ± 26.5	55.4 ± 16.5	<0.001	<0.001
Systolic SAP (mmHg)	113.3 ± 19.0	119.6 ± 15.0	111.5 ± 21.5	110.6 ± 12.1	109.1 ± 14.7	0.19	0.048
Mean SAP (mmHg)	85.3 ± 13.0	88.6 ± 14.3	84.5 ± 12.8	83.1 ± 12.2	84.0 ± 12.1	0.43	0.14
RA pressure (mmHg)	8 (6,11)	8 (6,11)	8 (6,11)	9 (8,11)	10 (8,13)	0.53	0.83
PAWP (mmHg)	11 (9,13)	12 (10,13)	10 (9,12)	12 (9,14)	12 (10,13)	0.104	0.03
CI (L/min·m^2^)	3.1 ± 1.0	3.1 ± 0.9	2.9 ± 1.0	3.4 ± 1.1	4.2 ± 0.9	0.004	0.48
PVR (WU)	13.2 ± 8.9	7.4 ± 6.2	15.4 ± 8.7	15.9 ± 9.6	10.5 ± 5.8	<0.001	<0.001
PVRI (WU·m^2^)	18.6 ± 13.4	10.9 ± 8.7	22.6 ± 14.0	19.4 ± 12.4	11.0 ± 5.0	<0.001	<0.001
PAC (mL/mmHg)	1.14 (0.75–1.91)	2.08 (1.07–1.96)	0.94 (0.71–1.54)	1.02 (0.65–1.90)	1.21 (0.81–2.59)	0.001	<0.001
Rp/Rs ratio	0.60 (0.34–0.93)	0.30 (0.18–0.49)	0.75 (0.50–1.03)	0.83 (0.42–1.08)	0.55 (0.45–1.03)	<0.001	<0.001
Time from CHD correction to diagnostic RHC (years)	3.6 (1.1–9.0)	1.2 (0.7–4.0)	6.3 (1.7–12.1)	3.6 (2.3–9.7)	2.0 (1.1–7.1)	<0.001	<0.001
Age at CHD correction (year)	11.5 (7.3,24.2)	27.7 (20.8,32.3)	10.1 (6.7,15.1)	8.5 (5.5,10.4)	9.9 (3.3,12.0)	<0.001	<0.001
PAH diagnosed by RHC before correction, *n* (%)	65 (51.2)	23 (74.2)	32 (43.8)	7 (46.7)	3 (37.5)	0.03	0.005
Qp/Qs ratio before correction	1.5 (1.2,1.9)	1.5 (1.2,1.8)	1.6 (1.2,2.1)	1.3 (1.0,1.8)	1.5 (1.1,1.9)	0.77	0.92
PVR before correction (WU)	9.5 ± 5.7	5.7 ± 2.0	10.6 ± 4.2	13.3 ± 6.6	17.6 ± 16.0	<0.001	<0.001
PVRI before correction (WU·m^2^)	11.5 ± 5.2	8.9 ± 3.1	12.2 ± 4.5	14.0 ± 5.4	18.4 ± 14.1	0.003	0.01
Rp/Rs ratio before correction	0.47 (0.35,0.69)	0.35 (0.28,0.40)	0.57 (0.41,0.71)	0.70 (0.59,0.75)	0.72 (0.49,1.09)	<0.001	<0.001
PAH therapies at last follow-up, *n* (%)	105 (81.9)	27 (87.1)	63 (86.3)	11 (73.3)	3 (37.5)	0.009	0.91
Combined therapies	55 (52.9)	11 (40.7)	36 (57.1)	6 (54.5)	2 (66.7)	0.53	0.36
Total follow-up time (patient-years)	593	121	339.5	84.2	47.8	0.30	0.32
Mortality during follow-up, *n* (% per patient-year)	12 (2.0)	1 (0.83)	7 (2.1)	3 (3.6)	1 (2.1)	0.42	0.40
Five-year survival rate (%)	89.6	95.0	93.0	70.1	85.7	0.50	0.32
Composite endpoint, *n* (% per patient-year)	35 (5.9)	3 (2.5)	24 (7.1)	7 (8.3)	1 (2.1)	0.01	0.09
Five-year event-free survival rate (%)	75.4	87.1	72.5	60.4	85.7	0.10	0.048

CHD, congenital heart disease; BSA, body surface area; WHO-FC, world health organization functional class; 6MWD, six-minute walk distance; Hb, hemoglobin; BNP, B-type natriuretic peptide; TRV, tricuspid regurgitation velocity; RV, right ventricle; PAWP: pulmonary artery wedge pressure; LV, left ventricle, TAPSE, tricuspid annular plane systolic excursion; PAP, pulmonary arterial pressure; TVR, tricuspid valve regurgitation; SvO₂, mixed venous saturation; SAP, systemic arterial pressure; RAP, right atrial pressure; CI, cardiac index; PVR, pulmonary vascular resistance; PVRI, indexed pulmonary vascular resistance; PAC, pulmonary artery capacitance; Rp/Rs, the ratio of pulmonary to systemic vascular resistance; PAH, pulmonary arterial hypertension; RHC, right heart catheterization; Qp/Qs, the ratio of pulmonary blood flow to systemic blood flow; WU, Wood unit

^a^Available in 46 patients

### CHD repair and timing of post-correction PAH diagnosis

At a median age of 11.5(7.3–24.2) years, 96(75.6%) patients had undergone surgical repair, and 31(24.4%) percutaneous device closure. On echocardiography prior to CHD correction, 107(84.3%) patients had signs of PH. Another 4(3.1%) patients (2 with small ASDs, 1 PDA and 1 PDA plus VSD) had no evidence of PH on pre-operative echocardiography and were operated at 3.0–5.6 years of age. In these 4 patients, a PAH diagnosis was established between 2 and 26 years after correction. There was no pre-operative hemodynamic or echocardiographic information in another 16(12.6%) patients.

RHC had been performed preoperatively in 66(52.0%) patients, all of whom were diagnosed with PAH: median PVR 9.5 ± 5.7 WU, PVRI 11.5 ± 5.2 WU.m^2^, with a ratio of pulmonary to systemic blood flow of 1.5(1.2–2.0). The ratio was >1.5 in 34(51.5%) patients, 1.2–1.5 in 20(30.3%), and <1.2 in 10 (15.2%), unavailable in 2(3%). Of patients with pre-operative RHCs, 1(1.5%) had a PVR <3 WU, 15(22.7%) had a PVR <5 WU, 1(1.5%) had a PVRI <4 WU.m^2^ and 16(24.2%) PVRI ≤8WU.m^2^.

The median time from CHD correction to post-operative diagnostic RHC was 3.6(1.2–8.9) years: 48(37.8%) patients were diagnosed with PAH <2 years from CHD correction, 24(18.9%) between 2–5 years, and 55(43.3%) ≥ 5 years. In 83(65.4%) patients, PH was detected during elective post-operative follow-up whereas in the remainder, PH was diagnosed either during pregnancy (4.7%), during non-cardiac procedures (1.6%), or on presentation to hospital with symptoms (28.3%): decreased exercise capacity (*n* = 15, 11.8%), dyspnea (*n* = 5, 3.9%), syncope (*n* = 10, 7.9%), hemoptysis (*n* = 1, 0.8%) and congestive heart failure (*n* = 5, 3.9%). At the time of diagnosis, the vast majority of patients (82.7%) were in World Health Organization functional class I-II while 17.3% were in functional class III-IV.

Patients who underwent CHD correction before 12 years of age had a longer time from repair to PAH diagnosis compared to those operated at an older age (7.2[2.3–12.1] years vs. 1.7[0.7–4.4] years), *P* < 0.001) and were more likely to be in World Health Organization functional class III-IV at enrolment (27.9% vs. 5.1%, *P* = 0.001). Patients who did not undergo RHC before CHD correction also had a longer time from operation to PAH diagnosis compared to those who underwent preoperative hemodynamic evaluation (8.4[3.6–12.3] years vs. 1.5[0.7–3.7] years), *P* < 0.001).

One half of patients (51.2%) were receiving targeted PAH treatment before the admission that confirmed the presence of post-operative PAH including 48(37.8%) as continuation of peri-operative PAH treatment: monotherapy in 46(70.8%) and combination therapy in 19(29.2%) patients. There were no significant differences in baseline WHO Functional Class III-IV (20% vs. 14.5%, *P* = 0.486) or PVR (12.5 ± 8.5 vs. 14.0 ± 9.3 WU, *P* = 0.35) between patients on treatment and those who were treatment-naïve at the baseline post-operative assessment. A ‘treat-and-repair’ strategy was used in 22(17.3%) patients. BNP was available in 125 patients at the time of diagnosis and was signiﬁcantly raised (>800 pg/mL) in 3 patients (2.4%), mildly raised (50–800 pg/mL) in 64(51.2%) and normal in 58(46.4%). By echocardiography, 64(52.5%) of 122 patients had a dilated RV with a basal RV to left ventricle (LV) ratio >1.0; the RV was significantly dilated with a ratio of RV/LV >1.5 in 19 patients (15.6%).

**Figure 2:**
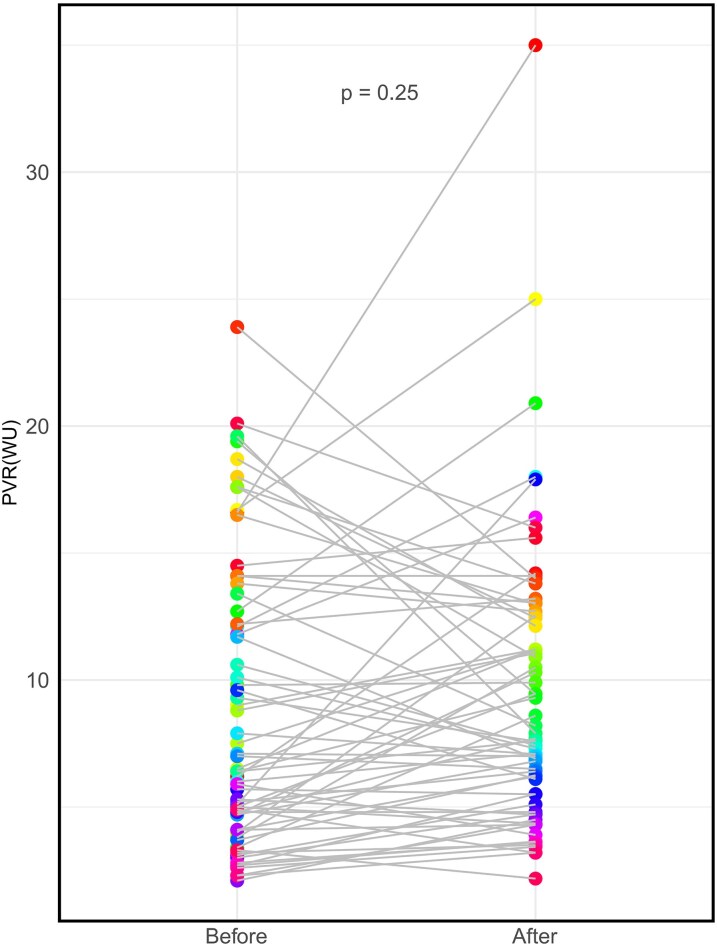
The changes in pulmonary vascular resistance (PVR) before and after congenital heart disease (CHD) correction in 66 patients who had pre repair hemodynamic evaluation.

On the diagnostic post-repair RHC, mean pulmonary arterial pressure was at supra-systemic levels in 34 patients (26.8%). A cardiac index <2.0 L/min/m^2^ was recorded in 17(13.4%) patients. Mean PVR and PVRI were 13.2 ± 8.9 WU and 18.6 ± 13.4 WU·m^2^ respectively, with 71(55.9%) patients having a PVR > 10 WU and 63(49.6%) having PVRI >14 WU·m^2^. The median PAC was 1.14(0.75–1.91) mL/mmHg. In 66 patients who had received preoperative RHC, there was a significant drop in mean pulmonary arterial pressure (65.6 ± 14.0 mmHg vs. 48.1 ± 20.3 mmHg, *P* < 0.001) after abolishing the shunt, but there was no significant change in PVR (9.4 ± 5.6 WU pre-repair vs. 8.7 ± 5.6 WU post-repair, *P* = 0.25) (*Figure 2*).

At diagnosis, patients with corrected simple pre-tricuspid shunts were older than patients with simple post-tricuspid shunts (28.2 ± 10.3 vs. 20.5 ± 9.0 years, *P* < 0.001), they were less likely to be in World Health Organization functional class III-IV (3.2% vs. 24.7%, *P* = 0.010), had a longer 6-minute walk distance (521 ± 114 vs. 467 ± 115 m, *P* = 0.04), lower mean pulmonary arterial pressure (41.9 ± 21.0 vs. 66.8 ± 24.3 mmHg, *P* < 0.001) and PVR (7.4 ± 6.2 vs. 15.4 ± 8.7 WU, *P* < 0.001), and lower RV free wall thickness (7[6–8] vs. 8[7–10] mm, *P* = 0.01) by echocardiography, but there was no difference in the ratio of RV/LV (0.90[0.83–1.23] vs. 1.05[0.87–1.32], *P* = 0.22, *Table 1*).

### Follow-up and survival analysis

**Figure 3:**
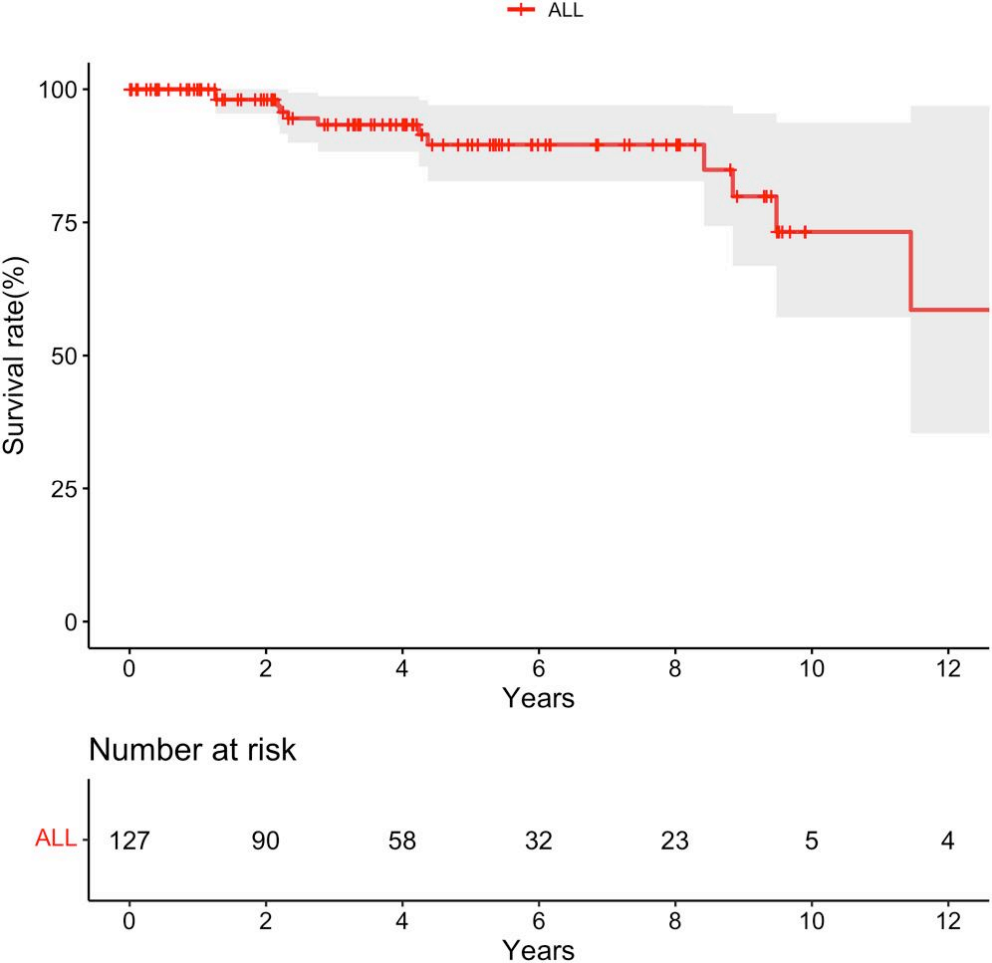
Kaplan-Meier survival curve of the 127 patients diagnosed with pulmonary arterial hypertension after congenital heart disease correction.

During a median follow-up of 4.0(2.0–6.4) years, 12 patients died (6 deaths due to right heart failure, 3 sudden deaths and 3 of unclear cause). Total follow-up time was 593 patient-years and the mortality was 2.0% per patient-year. Kaplan-Meier estimates of 3− and 5− survival were 93.3% (95%CI: 88.2–98.4%) and 89.6% (95%CI: 82.5–96.7%) (*Figure 3*). Five-year survival estimates for patients with a pre-tricuspid shunt, post-tricuspid shunt, combined shunts and complex shunts were 95.0%, 93.0%, 70.1% and 85.7%, respectively (Log-rank *P* = 0.50).

**Figure 4:**
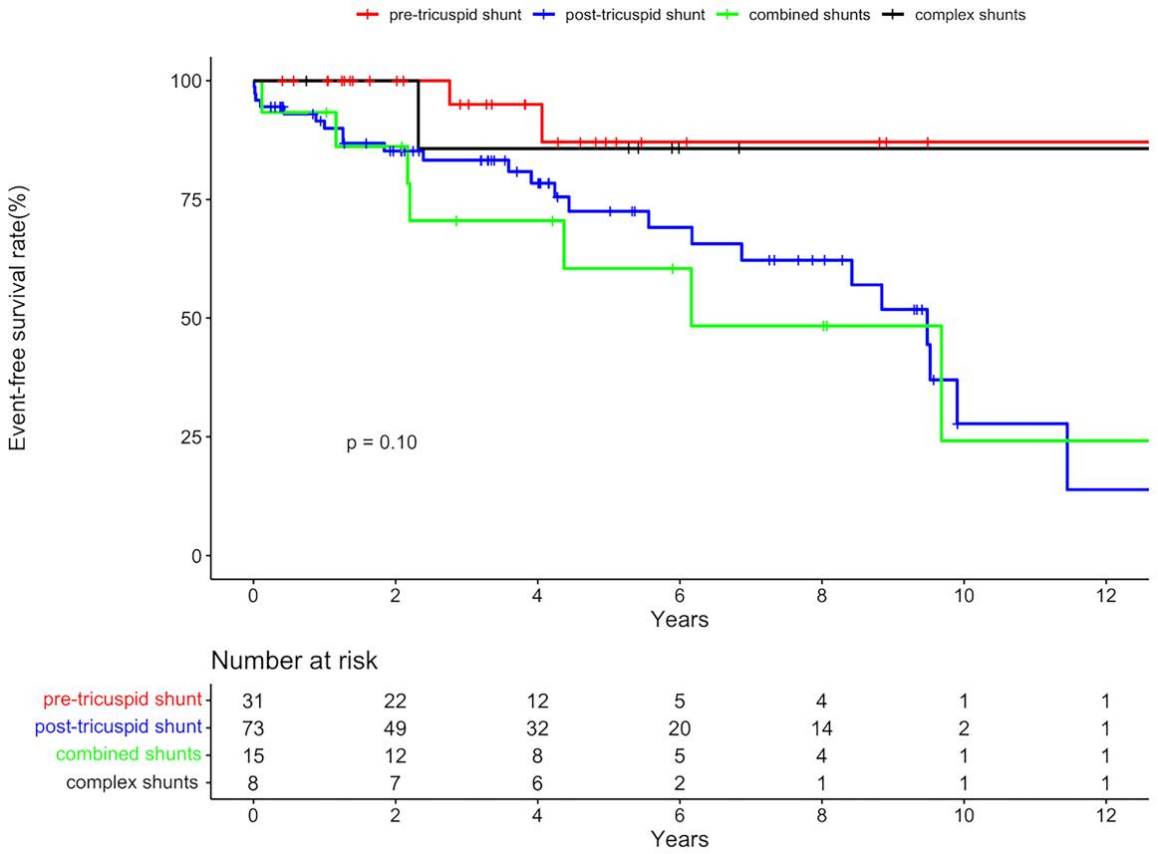
Survival curve depicting freedom from the combined mortality-morbidity endpoint for patients with underlying pre-tricuspid shunt, post-tricuspid shunt, combined shunts and complex shunts.

In total, 35 patients met the composite endpoint, with 29 patients admitted to hospital for worsening right heart failure, and 10 patients started on intravenous or subcutaneous Treprostinil. Kaplan-Meier estimates of 3− and 5− event-free survival were 84.4%(95%CI: 77.6–91.2%) and 75.4% (95%CI: 66.2–84.6%). Five-year event-free survival estimates for patients with a pre-tricuspid shunt, post-tricuspid shunt, combined shunts and complex shunts were 87.1%, 72.5%, 60.4% and 85.7%, respectively (Log-rank *P* = 0.10, *Figure 4*).

Other events included decreasing exercise capacity (*n* = 55,43.3%), syncope (*n* = 21,16.5%), hemoptysis (*n* = 5,3.9%), pulmonary embolism (*n* = 1,0.8%), atrial fibrillation/flutter (*n* = 4,3.1%) and ventricular tachycardia (*n* = 1,0.8%). At their last visit, 104(81.9%) patients were on targeted PAH therapy, including 49(47.1%) on monotherapy (endothelin receptor antagonists in 26, phosphodiesterase type-5 inhibitor in 19, beraprost sodium in 4), 40(38.5%) on dual-agent combination therapy and 15(14.4%) on triple-combination therapy; 3 patients(2.9%) were on continuous subcutaneous or intravenous treprostinil.

**Figure 5:**
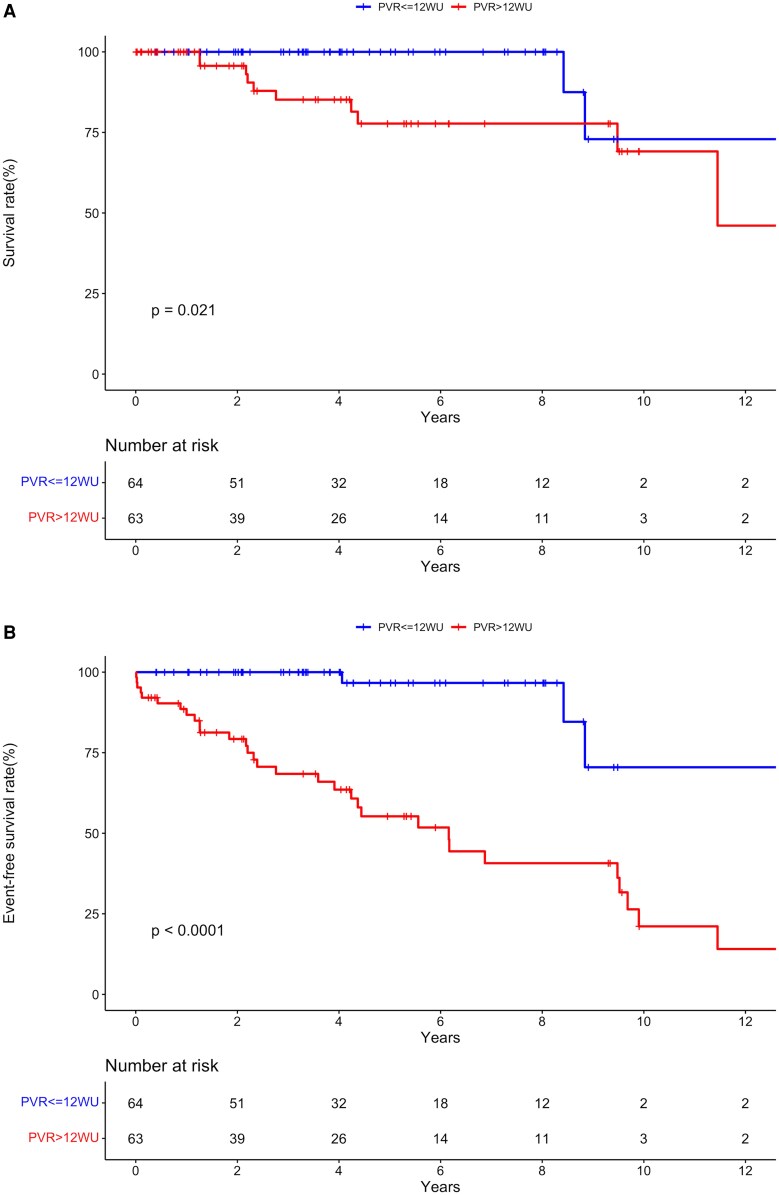
Clinical outcomes according to the median of pulmonary vascular resistance (PVR). (*A*) Survival. (*B*) Event-free survival.

On univariable analysis, 7 baseline variables were associated with all-cause death: BNP, 6-minute walk distance, peak tricuspid regurgitation velocity, mean pulmonary arterial pressure, right atrial pressure, PAC and PVR (*Table 2*, *Figure 5A*). Patients who fulfilled the composite endpoint were more likely to be in WHO Functional Class III-IV, had higher BNP, ratio of RV/LV, right atrial pressure, mean PAP and PVR, and a lower 6-minute walk distance, CI and PAC (*P* < 0.01 for all, *Figure 5B*). Older age at correction was associated with a lower risk of the composite endpoint (HR 0.922 per year, 95%CI: 0.877–0.969, *P* = 0.001). Three parameters (BNP, 6-minute walk distance and PVR) were entered into the multivariable Cox model for the composite endpoint, but only PVR remained in the model (hazard ratio 1.11, 95%CI 1.05–1.18, *P* < 0.001, *Table 3*). Indeed, patients with a PVR >10WU had a 14-fold increased risk of the composite endpoint (HR 14.23, 95%CI:3.4–59.48, *P* < 0.0001). Patients with a PVR > 12.5WU had a near 5-fold increased risk of death (HR 4.75, 95%CI:1.02–22.12, *P* = 0.047).

**Table 2:** Univariable analysis of risk factors for all-cause mortality

	Hazard ratio	95%CI	*P*-value
BNP (per 10 pg/mL)	1.014	1.004–1.025	0.008
6MWD (m)	0.993	0.988–0.999	0.01
Peak TRV (m/s)	2.620	1.238–5.544	0.01
Mean PAP (mmHg)	1.027	1.004–1.051	0.02
RAP (mmHg)	1.172	1.018–1.348	0.03
PVR (WU)	1.095	1.031–1.163	0.003
PAC (mL/mmHg)	0.189	0.040–0.890	0.035

BNP, B-type natriuretic peptide; 6MWD, six-minute walk distance; TRV, tricuspid regurgitation velocity; PAP, pulmonary arterial pressure; RAP, right atrial pressure; PVR, pulmonary vascular resistance; PVRI, indexed pulmonary vascular resistance; PAC, pulmonary artery capacitance.

**Table 3:** Univariable and stepwise multivariable cox hazard analysis of risk factors for the composite endpoint (35 events)

	Univariable analysis			Multivariable analysis		
	Hazard ratio	95%CI	*P*-value	Hazard ratio	95%CI	*P*-value
WHO-FC (III/IV vs. I/II)	5.220	2.577–10.574	<0.001			
BNP (per 10 pg/mL)	1.013	1.007–1.020	<0.001	1.007	0.997–1.017	0.190
6MWD (m)	0.993	0.990–0.996	<0.001	0.997	0.993–1.002	0.217
Peak TRV (m/s)	2.771	1.806–4.252	<0.001			
Ratio of RV/LV	6.452	3.400–12.245	<0.001			
Mean PAP (mmHg)	1.039	1.024–1.055	<0.001			
SvO_2_ (%)	0.898	0.858–0.939	<0.001			
RAP (mmHg)	1.136	1.037–1.245	0.006			
CI (L/min/m^2^)	0.503	0.336–0.753	0.001			
PVR (WU)	1.108	1.070–1.147	<0.001	**1**.**114**	**1.053–1.179**	**<0**.**001**
PVRI (WU·m^2^)	1.054	1.034–1.074	<0.001			
PAC (mL/mmHg)	0.094	0.030–0.301	<0.001			
Age at CHD correction (years)	0.922	0.877–0.969	0.001			
Time from CHD correction to diagnostic RHC (years)	1.110	1.060–1.163	<0.001			
Targeted PAH therapy	4.970	1.179–20.943	0.029			

WHO-FC, world health organization functional class; BNP, B-type natriuretic peptide; 6MWD, six-minute walk distance; TRV, tricuspid regurgitation velocity; RV, right ventricle; LV, left ventricle, PAP, pulmonary arterial pressure; SvO₂, mixed venous saturation; RAP, right atrial pressure; CI, cardiac index; PVR, pulmonary vascular resistance; PVRI, indexed pulmonary vascular resistance; CHD, congenital heart disease; RHC, right heart catheterization; PAH, pulmonary arterial hypertension. Statistical significance on multivariable analysis is indicated in bold font.

**Figure 6:**
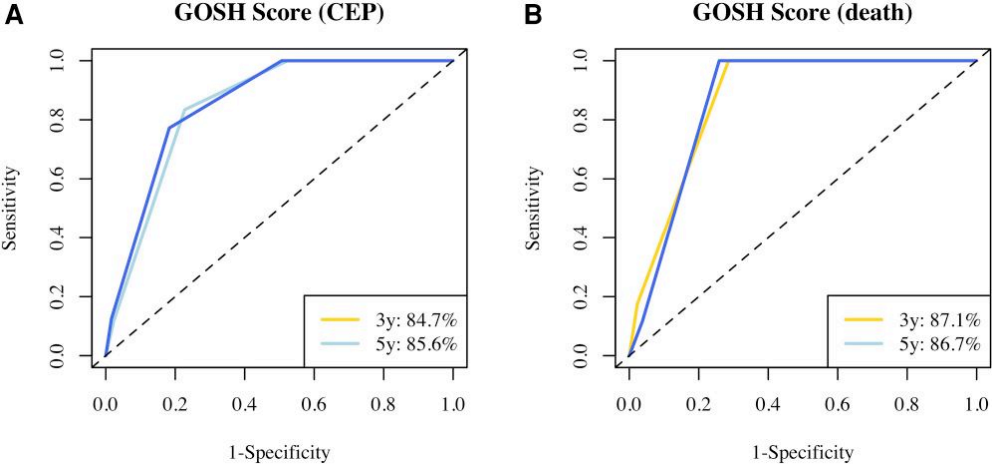
Time-dependent receiver operating characteristic curves(time ROC) and area under the curves for the combined endpoint (CEP, a) and death(b) at 3 and 5 years follow up.

The GOSH score was a strong predictor of both death (HR 3.17, 95%CI:1.5–6.71, *P* = 0.003) and the combined endpoint (HR 2.68, 95% CI:1.8–4, *P* < 0.0001) in our populations. On time-dependent Receiver-Operating Characteristic (time ROC) analysis, the score had an excellent discriminative power for both endpoints at 3 and 5 years: for death, AUC at 3 years was 0.871, 5 years 0.867; for the combined endpoint, AUC at 3 years was 0.847 and 5y 0.856 (*Figure 6*). There was no significant difference in AUC for either endpoint when adding BNP (>100 ng/L) to the GOSH score.

## Discussion

In this study, we report on a large population of patients with PAH-CHDcor managed in a single regional tertiary center. In this cohort, the presence of PAH was often established late after CHD repair, especially patients in whom PAH had not been detected pre-operatively, but also patients repaired ‘early’. PAH-CHDcor was associated with a high morbidity and mortality at medium-to-long-term follow-up, despite the widespread use of PAH therapies. In an exploratory analysis, the prognostic score developed by the GOSH had an excellent ability in identifying patients at risk of death or the morbidity-mortality endpoint. Close monitoring and lifelong follow-up of all patients after repair of CHD with a potential for pulmonary vascular disease is essential to ensure early diagnosis and proactive use of PAH therapies based on careful risk stratification.

Our data confirm the adverse prognosis of PAH-CHDcor. In our young population of patients with repaired CHD, a mortality of 2% per year, with an estimated 10% of patients dying at 5 years from diagnosis is concerning. The adverse prognosis of PAH-CHDcor is also reflected in the significant proportion of patients meeting the combined morbidity-mortality endpoint: one quarter at 5 years and two thirds by 10 years. Manes et al. and Alonso-Gonzales et al. have previously, shown that PAH-CHDcor carries a poor prognosis, albeit in older populations.^2,6^ The natural history and rate of progression of PAH depends on the ability of the RV to adapt to the increased afterload after closure of a congenital heart defect that could have acted as a ‘relief valve’ for a pressure-overloaded RV. Significant RV dilation and dysfunction were prevalent in our cohort, indicating that patients with post-repair PAH are likely to develop a ‘maladaptive’ RV phenotype, which is associated with a worse prognosis.^16,17^ When deciding on whether to close a defect in a patient with suspected or confirmed PAH, the detrimental short and long-term effects of the volume loading caused by the large shunt, are weighed against the perioperative risks and detrimental effects of PAH-CHDcor. Surgical delays in repairing post-tricuspid defects in our cohort reflect historical limitations in China's healthcare infrastructure, many of which have only recently been addressed. The threshold for repairing CHD has lowered in recent years, with better perioperative care and the availability of percutaneous options and PAH therapies.^18^ However, the significant medium-to-long morbidity and mortality observed in our population calls for caution, careful characterization and close monitoring of such patients.

Understanding the different modes of presentation of PAH-CHDcor is important in optimizing management and avoiding late diagnosis, which can be very detrimental and contribute to the significant morbidity and mortality. PAH may be detected pre-operatively and persist after CHD correction, or can be identified *de novo,* early or late after CHD correction The vast majority of patients with PAH-CHDcor had evidence of PH on preoperative echo, yet in only one half was this deemed severe enough to require cardiac catheterization before surgery of intervention. While cardiac catheterization is essential for establishing the presence and severity of PAH, it does carry risks, especially in younger children with comorbidity^19^; in such cases, a decision to operate may be taken based on the clinical presentation and imaging information, especially if PH is deemed mild on echo, carefully weighing risks vs. benefits. In all cases with suspected or confirmed PAH prior to CHD repair, careful perioperative management and post-operative hemodynamic evaluation is required to establish the diagnosis of PAH-CHDcor and decide on PAH therapy and follow-up.

There was a smaller group of patients without evidence of PAH before CHD closure, in whom PAH developed years after repair. In 4 patients, the congenital shunt had been small, and PAH may be deemed ‘coincidental’ to the CHD, rather than caused by it; in such patients, experts believe that there may be a genetic or other intrinsic predisposition of the pulmonary circulation towards the development of PAH, that may be triggered by the shunt.^20–22^ The role of genetics in the development and progression of PAH in patients with CHD, and its influence on operability decisions remains unknown.

Late diagnosis is detrimental to all PAH patients, including those with PAH-CHDcor. A significant proportion of our population were diagnosed only after developing symptoms, sometimes in adverse circumstances, e.g. during pregnancy or when overt congestive heart failure or hemoptysis occurred. In our population, patients with a longer interval from repair to diagnostic RHC tended to present in worse functional class, plausibly due to loss-to-specialist follow-up and/or symptom underreporting. The timing of presentation with PAH-CHDcor may be pathophysiologically informative: early post-operative PAH likely reflects pre-existing disease or peri-operative remodelling, whereas PAH arising years later may suggest a different pathogenetic process, which may be progressive and is often associated with a delay in diagnosis, hence presenting as advanced disease. CHD patients are known to under-report symptoms, which would explain the low functional class in a population with a significantly raised PVR. BNP was helpful in predicting outcome, but was normal in almost one half of patients at the time of diagnosis, hence cannot be relied upon to detect PAH-CHDcor. The echocardiographic detection of PH in CHD requires expertise to overcome confounders, such as residual lesions (e.g. valvar or ventricular dysfunction), or the absence of a sufficient tricuspid regurgitation Doppler signal.^23^ It is a worry that, compared with adolescents and adult patients, children were more likely to be underdiagnosed or diagnosed late, with a more severe clinical presentation at diagnosis. Outcomes can improve with better education of patients and their families on the importance of regular, lifelong follow-up and the need to seek medical attention when they develop new and/or concerning symptoms.^1,18^

Our data suggest that the RHC is not only essential in confirming the diagnosis of PAH pre and post-closure of the defect, but can also provide important prognostic information in this cohort. PVR is the single more important hemodynamic parameter in PAH-CHD, most closely reflecting the severity of PVD. Interestingly, in patients with both pre and post-repair cardiac catheterization, there was no change in average PVR (despite many patients receiving PAH therapies at the time of PAH-CHDcor diagnosis), while the drop in PA pressures is likely to reflect the reduction in pulmonary blood flow when the shunt is abolished. This underlines the importance of not relying to echocardiography alone to assess the change in hemodynamics post-repair, but also suggests that abolishing the shunt has little effect on the severity of PVD.

We were able to externally validate the previously reported GOSH score for patients diagnosed with PAH-CHDcor, which was highly discriminative of both death and the combined morbidity-mortality endpoint in our population. Given that the GOSH score was derived from an exclusively paediatric cohort, its use in our population was deemed exploratory. The score, however, includes readily available clinical parameters, including PVR, which was the only independent predictor of the morbidity-mortality endpoint and a univariable predictor of mortality (multivariable survival analysis was not possible due to the low absolute number of deaths). Right atrial pressure was also an univariable predictor of mortality and morbidity and is included in the risk stratification algorithm of the international PAH guidelines for PAH.^13^ Cardiac catheterization should, therefore, be routinely performed in the weeks or months after repair in patients with confirmed or suspected preoperative PAH, and in any patient with suspected PH at any point after CHD correction. Indeed, given that 43% were diagnosed >5 years after repair and that PVR at diagnosis was the principal predictor of outcome, the results of this study support lifelong systematic surveillance after CHD repair; in patients with pre-operative PH or high-risk anatomy there is a role for routine post-repair right heart catheterization within months to document haemodynamics and guide therapy, and there should be a low threshold for repeat invasive reassessment when appropriate.

A distinction should be made between pre- and post-tricuspid shunts in terms of their potential for triggering PVD. Large post-tricuspid defects should be repaired early, ideally within 6 months of age, to avoid the development of PVD.^24^ In our cohort, two-thirds of patients had a post-tricuspid shunt, yet the age at repair was well beyond 1 year in most (median age at surgery or intervention 11.5 years), which would explain the large proportion of patients with signs of PH on preoperative echo, confirmed in many on cardiac catheterization. Pre-tricuspid shunts, e.g. ASDs, cause volume but not pressure overload of the pulmonary circulation and are not expected to trigger PVD, even when large. This suggests that additional factors, including genetic predisposition, contribute to the development of PVD at a later age.^25^ In our cohort, these patients were older, had a lower PVR and were functionally less impaired than patients with post-tricuspid shunts, even though they often present with larger and more impaired RVs than patients with post-tricuspid shunts. Recent guidelines advocate for fenestrated closure for patients with an ASD and high baseline PVR that drops to <5WU after a course of PAH therapy (‘treat-and-repair’ strategy).^26,27^ No recommendations for a treat-and-repair approach and/or fenestrated closure exist for patients with post-tricuspid shunts.

Over 80% of patients received targeted PAH therapies in our study, and over half were on combination therapy. Patients with PAH-CHDcor have been included in randomized trials of PAH therapies, together with idiopathic and other PAH, providing convincing evidence on the use of single and early sequential combination therapy to improve exercise capacity and clinical outcomes.^28,29^ The management of all PAH patients should rely on regular risk-stratification, though the risk algorithm proposed by the guidelines has not been validated in PAH-CHDcor.^13^ Our data support the use of PVR at the time of diagnosis as an important prognostic parameter (albeit with a different cutoff), that can be used to identify patients at greater risk of adverse outcome who are more likely to benefit from aggressive treatment and close follow-up. Our study was not designed to assess the efficacy of PAH therapy in this setting, which is already supported by published data, including randomized controlled trials. In line with clinical guidelines, patients with PAH should be assessed for early combination therapy, further escalation when appropriate and timely consideration of reverse Potts shunt or lung transplantation when risk remains high despite optimal medical therapy^13^; these advanced surgical strategies were not available during the study period.

There are several limitations in this study. Firstly, this was a retrospective, single-center study focusing on patients with evidence of PAH. Patients who died around or early after repair, or without a diagnostic RHC, were not included, potentially introducing immortal-time bias and prohibiting us from using our data to identify indices of operability. Most of our patients were children and young adults, and may have a different clinical picture and outcome compared to older adults. Few patients in our cohort had complex shunts, a widely heterogeneous population in terms of anatomy and pathophysiology. The small sample size made comparison between all anatomical groups difficult, and patients with palliated univentricular circulations, e.g., after Fontan-type surgery, were not included in this study. Systematic cardiopulmonary exercise testing was not undertaken. Finally, long-term (≥10-year) survival estimates present a high level of uncertainty due to limited numbers-at-risk in this heterogeneous cohort; multivariable modelling, therefore, focused on the composite endpoint. While our follow-up time was long, it may not have captured very long-term adverse effects of CHD repair in patients with established PVD, which may require several years or even decades to manifest.

### Conclusion

In this study, we report on a large cohort of patients with PAH-CHDcor, highlighting a significantly adverse clinical outcome at mid-to-long-term follow-up, especially in those with a higher PVR on diagnostic catheterization. All patients who undergo closure of a congenital heart defect with the potential to trigger pulmonary vascular disease should receive life-long surveillance in a specialist center, with particular attention to patients with evidence of PH prior to repair who should be considered for invasive assessment soon after defect closure. Early diagnosis allows careful risk stratification using the GOSH score, now externally validated in our population, and ensures specialist care and evidence-based PAH therapy that can improve the outcome of this fragile population of patients with PAH-CHDcor.

## Data Availability

To minimize the possibility of unintentionally sharing information that can be used to re-identify private information, in line with the conditions of the ethical approval, patient-level data are not available for use outside of this study.
